# Thyroid hormone resistance syndrome caused by heterozygous A317T mutation in thyroid hormone receptor β gene: Report of one Chinese pedigree and review of the literature

**DOI:** 10.1097/MD.0000000000004415

**Published:** 2016-08-19

**Authors:** Qing-Hua Guo, Bao-An Wang, Chen-Zhi Wang, Min Wang, Ju-Ming Lu, Zhao-Hui Lv, Yi-Ming Mu

**Affiliations:** a Department of Endocrinology, Chinese PLA General Hospital, Beijing; b Department of Endocrinology, Hainan Branch of Chinese PLA General Hospital, Sanya, Hainan; c Domestic Inpatient Department of HMI, Chinese PLA General Hospital, Beijing, China.

**Keywords:** Chinese, pituitary thyroid hormone resistance syndrome, point mutation, thyroid hormone receptor β (THR β), thyroid hormone resistance syndrome

## Abstract

**Background:**

Thyroid hormone resistance syndrome (THRS) is a rare disorder with increased concentrations of free thyroxine (FT4) and triiodothyronine (FT3), but normal or slightly increased thyroid-stimulating hormone (TSH). The mutations in the thyroid hormone receptor β (THRβ) gene are thought to be the main pathogenesis.

**Objectives:**

The aims of this study were to present 1 pedigree of Chinese THRS, summarize their clinical characteristics, and analyze the gene mutation.

**Methods:**

The clinical characteristics and thyroid function of the proband and his family members were collected. Gene mutations were analyzed by DNA sequencing.

**Results:**

The proband and his mother exhibited symptoms of hyperthyroidism, such as palpitations, heat intolerance, and perspiration. The mother also had atrial fibrillation. The rest of the kindred did not display clinical manifestations of hyper- or hypothyroidism. DNA sequencing revealed a heterozygous G>A missense mutation at position 949 in Exon 9 of THRβ both in the patient and his mother, which led to the transition from alanine to threonine at position 317 of THRβ protein (A317T), whereas the rest of the kindred did not share this mutation. The proband and his mother were diagnosed with pituitary resistance to thyroid hormone. Oral administration of methimazole was stopped and β-receptor blockers were administrated.

**Conclusions:**

We present 1 pedigree of THRS with heterozygous A317T mutation in THRβ gene in the proband and his mother, which is the first reported mutation in Chinese and provides a comprehensive review of available literature.

## 1 Introduction

Thyroid hormone resistance syndrome (THRS)^[1]^ is a rare autosomal dominant or recessive disorder that occurs in familial and sporadic cases. The clinical manifestation includes reduced responsiveness of the targeted tissues (pituitary and/or surrounding) to thyroid hormone, which leads to increased concentrations of free thyroxine (FT_4_) and triiodothyronine (FT_3_), but normal or slightly increased serum thyrotropin (thyroid-stimulating hormone [TSH]) levels that result in the appearance of symptoms of hyper- or hypothyroidism. The incidence of THRS is approximately 1:40,000.^[2]^ Since the first report of THRS by Refetoff et al^[3]^ in 1967, >300 pedigrees and 1000 cases have been reported abroad.^[4–7,24]^

In recent years, dozens of cases have been documented in China,^[8–11]^ including both familial and sporadic ones. However, most of the cases demonstrate systemic hormone resistance, and there is limited information on selective pituitary resistance to thyroid hormone. Most THRS cases are caused by genetic mutations in the thyroid hormone receptor β (THRβ) gene,^[1,13–14]^ but in some cases, no mutations have been harbored.^[11,15]^ In this study, we examined the mutations in THRβ gene in a pedigree with selective pituitary resistance to thyroid hormone syndrome diagnosed at Chinese PLA General Hospital.

## 2 Case reports

### 2.1 Proband (III:1)

A 24-years-old male came for diagnosis because of heat intolerance, perspiration, palpitations, insatiable appetite, and constant hunger for 3 years. Previous medical records showed abnormal thyroid functions, indicated by increased serum FT_3_, serum FT_4_, and serum total thyroxine (TT_4_) levels, whereas those of TSH remained in the normal range. The results from previous blood tests were as follows: serum total triiodotyhronine (TT_3_) 3.20 ng/dL (reference values: 0.66–1.92), TT_4_ 23.12 μg/dL (reference values: 4.30–12.5), FT_3_ 9.49 pg/mL (reference values: 1.8–4.1), FT_4_ 3.56 ng/dL (reference values: 0.81–1.89), TSH 2.87 μIU/mL (reference values: 0.38–4.3); alanine aminotranferease (ALT) 173 U/L, and aspartate aminotransferase (AST) 87 U/L. Oral administration of methimazole (5–10 mg/d) and liver protection drugs for 2 to 4 weeks did not improve thyroid function or hyperthyroidism symptoms. The proband visited several hospitals and the methimazole dosage had been repeatedly adjusted (2.5–10 mg/d). However, the thyroid function was not restored. In his history, the proband was born full-term, with a normal IQ and postnatal growth and development. Family history includes the proband's mother having a medical history of hyperthyroidism for around 30 years. Physical examination includes: body temperature 36.5°C; respiration 18 times/min; heart rate 86 beats/min; blood pressure 120/80 mmHg; height 168 cm; body weight 60 kg; normal development, medium nutrition, damp skin, negative eye symptoms; thyroid enlargement (degree II), soft and no nodules, no tenderness, tremor or vascular murmur; heart rate 86 beats/min, no arrhythmias, with hands shaking (+); no edema in lower extremities. Laboratory findings include: FT_3_ 9.02 pmol/L (reference 2.76–6.30), TT_3_ 3.32 nmol/L (reference 1.01–2.95), FT_4_ 39.01 pmol/L (reference 10.4–24.3), TT_4_ 223.5 nmol/L (reference 55.3–160.8), TSH 0.98 mU/L (reference 0.35–5.5), thyroglobulin antibody (TGAb) <60 (reference<60 IU/mL), thyroid peroxidase antibody (TPOAb) <60 (reference <60 IU/mL), thyrotropin receptor antibody (TRAb) <0.3 (reference <60 IU/mL). The results of complete blood count, routine urinalysis, blood glucose, and renal function were within normal ranges. Gonadal function, evaluated by the levels of follicle-stimulating hormone (FSH), luteinizing hormone (LH), estrodiol, progesterone, testosterone, and prolactin (PRL), was normal. The ultrasound examination of the thyroid gland indicated uneven echoes and strong signals of blood flow within the gland. Iodine^131^ uptake rate was 46.38% at 4 hours and 67.14% at 28 hours. The scan of the somatostatin receptor revealed an enlarged thyroid gland and increased uptake, which was in line with hyperthyroidism symptoms. The rest of the tissue was not seen in the image. MRI scanning and dynamic contrast-enhanced MRI displayed no abnormalities in the pituitary.

### 2.2 Kindreds

**Figure 1:**
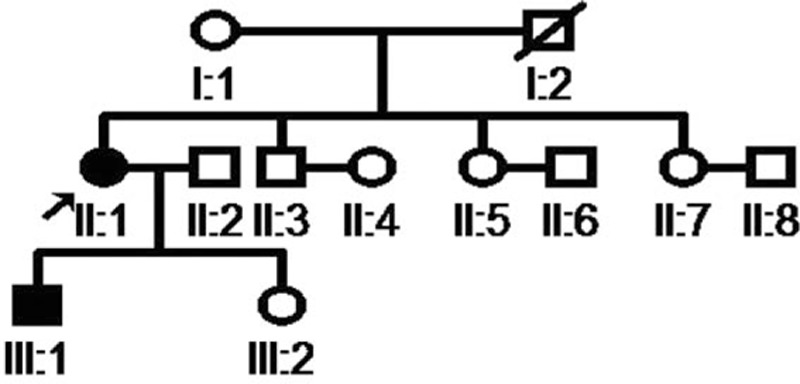
The pedigree of thyroid hormone resistance syndrome.

Three generations of the pedigree aged 18 to 90 years are presented in Figure 1. The family had no history of consanguineous marriage. The proband (III:1) had a sister (III:2) with no history and symptoms of hyperthyroidism. The proband's mother (II:1), 50 years’ old, had experienced symptoms of hyperthyroidism, such as palpitation, heat intolerance, perspiration, and tremor for around 30 years. Multiple tests confirmed that her FT_3_, TT_3_, FT_4_, and TT_4_ levels were higher than the normal, whereas the TSH level was within the normal range. TRAb was <0.3 IU/mL (reference <60). She was diagnosed with hyperthyroidism by other hospitals and took methimazole orally. However, her symptoms were not relieved, and biochemical test indices were not improved. Recently, she was diagnosed with atrial fibrillation. Physical examination includes: height 152 cm, body weight 65 kg, with normal development. No edema was observed in eyelids and no exophthalmos. Thyroid was slightly enlarged, soft, no tenderness and nodules; no vascular murmur was detected. The resting heart rate was 88 beats/min with arrhythmias. No pathological murmur was detected in any of the valve areas. Hands shaking (+) was observed, but no edema was found in lower extremities. EEG suggested atrial fibrillation. Ultrasound of thyroid gland revealed diffuse swelling and rich signals of blood flow. Pituitary MRI was negative. The proband's father (II:2) showed no symptoms of hyperthyroidism; the 3 siblings of the proband's mother (II:1) (II:3, II:5, II:7) and the proband's maternal grandmother (I: 1) did not have symptoms of hyperthyroidism either. The proband's maternal grandfather died of pneumonia in his 70s.

## 3 Methods

### 3.1 Hormone measurement

Venous blood (4 mL) was drawn from the proband and his kindred and centrifuged at 3000 rpm for 7 min. The sera were analyzed for thyroid function parameters using chemiluminescence immunoassay (Siemens, ADVIA Centaur). The intra- and interbatch variation of the assays were both <5%.

### 3.2 Extraction of genomic DNA from peripheral blood

Venous blood (8 mL) was drawn from the proband and his kindred with EDTA added as anticoagulant and centrifuged at 3000 rpm for 7 minutes. White blood cells were isolated, and DNA was extracted using the standard phenol-chloroform method. The purity and concentration of each DNA sample were determined by UV spectrophotometry.

### 3.3 Amplification and sequencing of *THRβ* gene

PCR-DNA sequencing was applied to examine all 10 exons of THRβ gene. According to THRβ gene sequence provided by NCBI (NG_009159.1), primers covering each of the 10 exons and adjacent introns were designed by primer premier software. The primer sequences, PCR product size, and annealing temperature were shown in Table 1. Primer synthesis, PCR reaction, and sequencing were performed by Taihe China-US Biotechnology Co, Ltd (Beijing, China): Taihe Taq polymerase; ABI GeneAmp9700 PCR system; ABI 3730XL DNA analyzer. Sequencher software was used to align the sequencing results against NG_009159.1 sequence to identify the mutation sites.

**Table 1:**
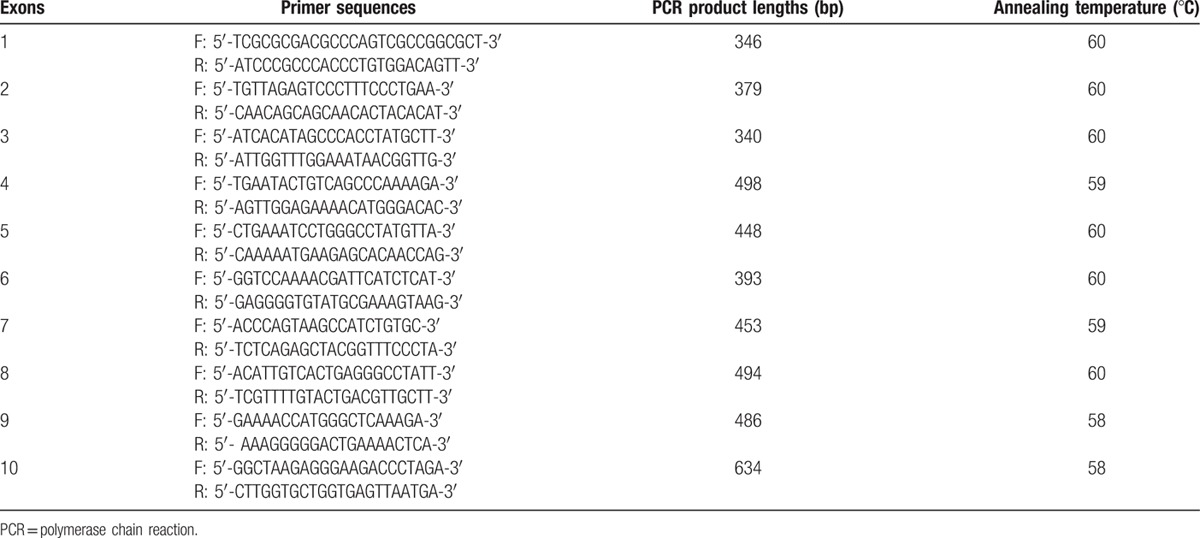
Primer sequences and PCR product length of 10 exons of *THRβ* gene.

This study was approved by the Ethics Committee of the Chinese PLA General Hospital. Informed consent from the patient and his family was obtained before the study.

## 4 Results

### 4.1 The thyroid function of the kindred

The thyroid functions of the proband's kindred were summarized in Table 2. Consistent with clinical manifestation of hyperthyroidism in the proband and his mother, their FT_3_, TT_3_, FT_4_, and TT_4_ were higher than normal, whereas TSH remained within the normal range. The rest of the family had normal thyroid functions.

**Table 2:**
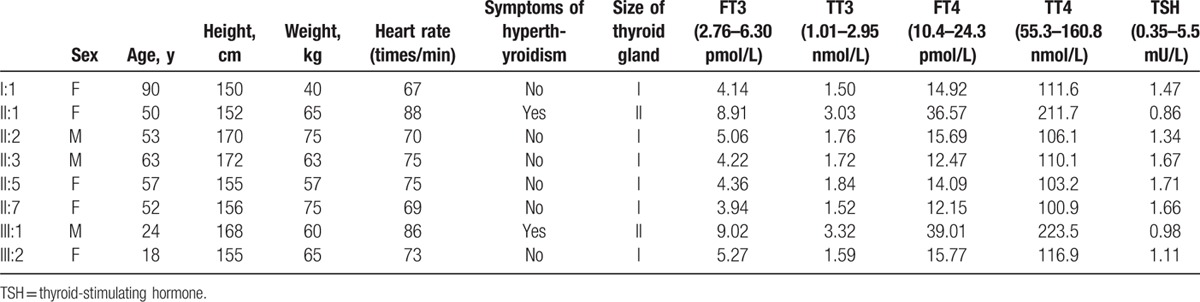
Thyroid hormone levels of the proband's kindred.

### 4.2 Results of *THRβ* gene exon sequencing

**Figure 2:**
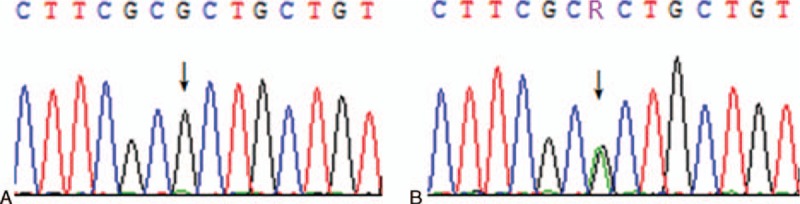
Partial sequencing result of Exon 9 of the thyroid hormone receptor β (*THRβ)* gene. (A) normal; (B) proband and his mother (arrows point to the base of the mutation).

We sequenced Exon 1 to 10 of THRβ for the proband and found a heterozygous G>A missense mutation at nucleotide position 949 in Exon 9 (Fig. 2B), which changed the trinucleotide codon GCT into ACT and led to a transition from Alanine to Threonine at position 317 of the gene-coding product (A317T). We also sequenced Exon 9 for the other kindred and found that the proband's mother shared the same mutation, whereas the rest of the family did not harbor it (Fig. 2A).

### 4.3 Current treatment and follow-up

We did not found any improvement in the thyroid function or hyperthyroidism symptoms in the patients and his mother by oral administration of methimazole. After the diagnosis of pituitary resistance to thyroid hormone in these patients was confirmed by the clinical features, laboratory findings, and gene mutation analysis, we stopped the oral administration of methimazole and administered β-receptor blockers to the patient and his mother. One month later, the clinical symptoms of the patients were greatly relieved.

## 5 Discussion

THRS is a common autosomal dominant or recessive genetic disorder. However, approximately 15% of the cases are sporadic.^[6]^ According to the sites where thyroid hormone resistance occurs, THRS can be categorized into global resistance to thyroid hormone (GRTH), pituitary resistance to thyroid hormone (PRTH), and peripheral resistance to thyroid hormone (PrRTH). Approximately 80% of the patients with THRS belong to the GRTH type. PRTH is not widespread, whereas only a few cases of PrRTH have been reported.^[1]^

Owing to the diverse clinical manifestations^[24]^ and the insufficient knowledge, THRS tends to be overlooked or misdiagnosed. Generally speaking, most patients with GRTH do not have significant clinical manifestation: patients with complete resistance exhibit severe clinical symptoms, whereas those with incomplete resistance display mild or no symptoms at all. Patients with PRTH demonstrate mainly mild to moderate hyperthyroidism, without exophthalmos or anterior tibial myxedema. PrRTH is exceedingly rare and is mainly manifested as hypothyroidism. The clinical features are: thyroid goiter; hypothyroidism or normal thyroid functions; increased serum thyroid hormone levels accompanied by normal or increased TSH level.

The proband of this pedigree was a 24-year-old male with clinical symptoms of hyperthyroidism. No exophthalmos or anterior tibial myxedema was observed. His FT_3_ and FT_4_ values were higher than the normal, whereas that of TSH was within the normal range, which then excluded the possibility of hyperthyroidism caused by hyperactivity of the thyroid gland itself. In addition, the results from pituitary MRI were negative, and the proband's mother had similar symptoms and thyroid hormone levels. Therefore, the proband was considered to have pituitary resistance to thyroid hormone. Further genetic analysis revealed that both the proband and his mother had a heterozygous missense mutation in Exon 9 of *THRβ* gene, which led to a transition from alanine to threonine at position 317 (A317T). This A317T mutation was one of the hotspot mutation sites of PRTH identified abroad,^[18]^ whereas domestically, this mutation has not been reported to cause PRTH. The fact that the other family members manifested no symptoms of hyperthyroidism or mutations in *THRβ* gene further confirmed the proband's diagnosis of PRTH.

THRβ gene mutation is considered the most important cause of THRS.^[13,19]^ Thyroid hormone receptor contains THRα and β, which mainly refers to T3 receptor. It belongs to the nuclear receptor superfamily and possesses 4 functional domains from N-terminus to C-terminus: transcription activation domain that participates in gene transcription activation; DNA-binding domain that binds to DNA and is involved in receptor dimerization; hinge region; ligand-binding domain that binds to ligands and coregulators. *THRβ* gene produces 2 isoforms, THRβ1 and THRβ2, by different transcription start points. THRβ1 contains 10 exons that encode a product with a length of 461 amino acid residues, among which amino acid residues 178 to 461, encoded by Exon 7 to 10, compose the C-terminal ligand-binding domain and part of the hinge region. THRβ2 has 15 more residues than THRβ1 at the amino-terminus. Around 80% of THRS cases are caused by THRβ gene, and no THRβ mutation is found in 10% to 15% of THRS cases.^[1,13,16]^ Recently, mutations in human THRα have been reported as possible causes of THRS. ^[17]^

Up to now, all THRβ mutations have been reportedly located in 3 hotspot regions between Exon 7 and 10 (234–282, 310–353, and 429–461).^[14,18,20–24]^ Only A229T, R243W, and R243Q are located in Exon 7, whereas most of the mutations are located in Exon 9 and 10. No mutation was found in the amino-terminus, the DNA-binding domain. The hotspot regions correspond to the ligand-binding pocket in the 3D structure, and the mutations in this area can lead to a loss of binding or reduced binding between the receptor and thyroid hormone, which eventually results in insensitiveness or resistance to the thyroid hormone. Moreover, mutated receptors are dominant negative and are capable of blocking the function of wild-type receptors. For example, they can suppress the normal transcriptional activity by inhibition of the wild-type T3 receptor and competition it for binding the TH-response elements (TREs) in the promoter regions of the effector genes.^[22,25,26]^

Mutations have been now identified in over 300 families, most of which have single-nucleotide substitutions, resulting in 1 amino acid replacement and in a few truncated molecules. Twenty other families have nucleotide deletions, insertions, or duplications, some producing frame shifts that create nonsense proteins. From the 171 different mutations identified, the same mutation may be carried by several different families. Diverse mutations have resulted different clinical manifestations. A summary of main thyroid hormone receptor gene mutations and clinical manifestations was displayed in Table 3.^[4,5,7,23,24,27–32]^ THRβ R338W has been identified in 33 unrelated families. Mutations have also produced different amino acids in the same codon. Seven such different substitutions were identified in codon 453 (P453T, S, A, N, Y, H, L).^[4–7,24]^

**Table 3:**
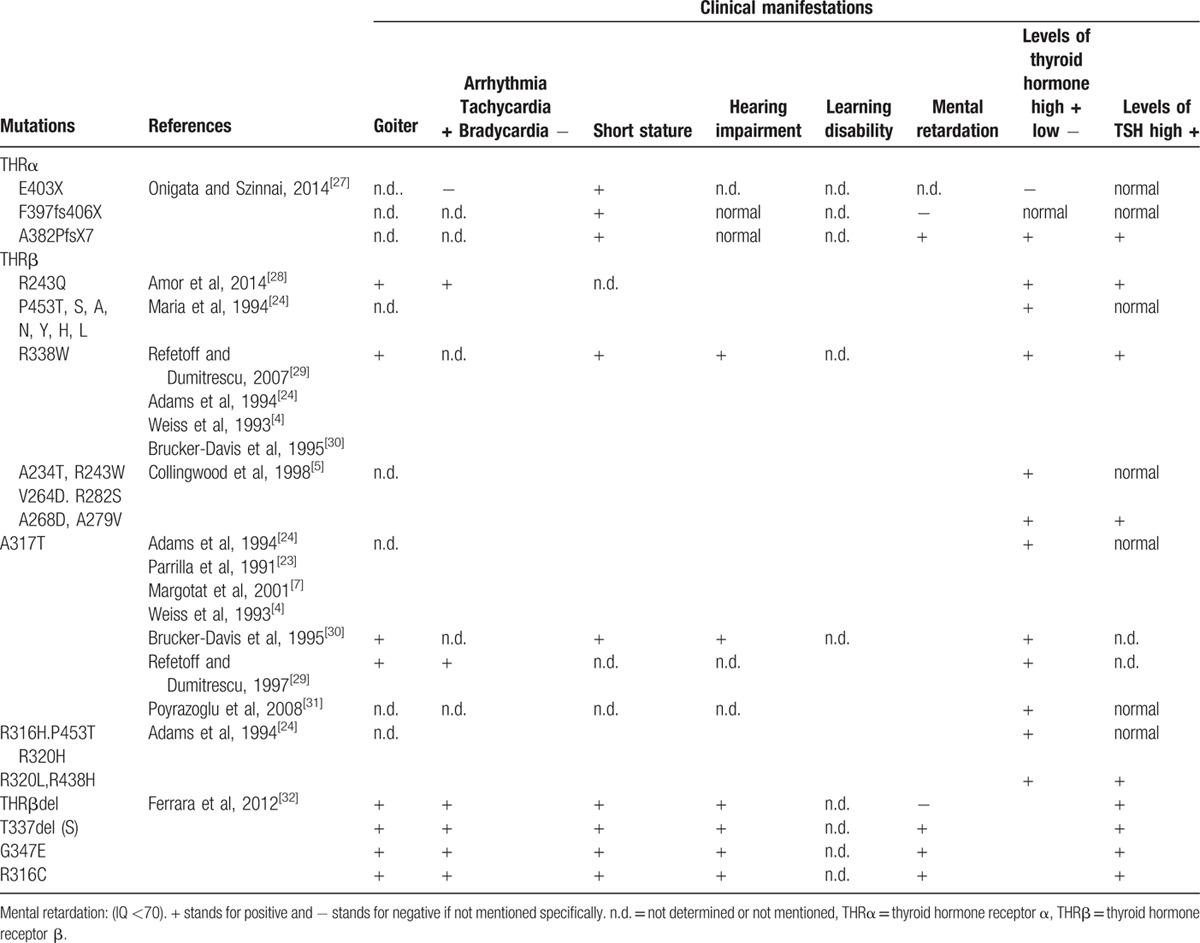
Thyroid hormone receptor mutations and their related clinical manifestations.

The 5 mutations that are most frequently reported abroad include R338W, A317T, R438H, R243Q, and P453T.^[18,33]^ Domestically, point mutations, such as P453A,^[8]^H435L,^[9]^ and V458A^[10]^ have been reported. The A317T mutation in Exon 9 of THRβ gene identified in this pedigree with PRTH is one of the hotspot mutations detected abroad,^[23–24,31,34,35]^ which has not been found in China before.

According to literature, the A317T mutation in *THRβ* gene was located in the ligand-binding domain.^[23–24,35]^ Whether in familial or sporadic cases, the T3-binding affinity of receptors with A317T mutation was only 12% to 20% of that binding affinity of wild-type receptor. Therefore, this mutation reduced the binding between the receptor and T3 and caused hormone resistance.

Interestingly, only a few among many THRβ mutations that cause THRS, such as R429Q, R338L, and R338W, can specifically lead to selective PRTH. The rest of the mutations do not result in consistent clinical manifestations.^[23–24]^ The same mutation in one case may cause PRTH, but in another it may induce GRTH.^[36]^ In this pedigree with PRTH, we identified the mutation A317T, which was reported in the literature to cause mainly GRTH, suggesting that the phenotype of thyroid hormone resistance was not solely dependent on the mutation site.

Another possible explanation is that different phenotypes produced by the same genetic mutation are associated with the variations in the distribution of receptors. THR contains subtypes, including THRα_1_, THRα_2_, THRβ_1_, and THRβ_2_. THRα_1_ and THRβ_1_ are ubiquitously expressed, whereas THRβ_2_ is expressed only in pituitary, hypothalamus, retina, and inner ear. If THRβ mutation affects mainly THRβ_1_, then the phenotype is GRTH; if the mutation influences predominantly THRβ_2_, then the phenotype is PRTH. It has been reported that selective PRTH was caused by mutations in *THRβ*_*2*_ gene.^[36]^

Approximately 10% to 15% of the patients diagnosed with THRS had no mutations in *THRβ* gene.^[7,11–13,16]^ Previously, it was speculated that this was associated with post-receptor defects, abnormality in coregulators, lack of type II 5′-deiodinase in pituitary, etc.^[37]^ However, recent studies revealed that mutations in *THRα* gene can also cause THRS.^[38]^ Therefore, whether those reported patients diagnosed with THRS but without *THRβ* gene mutation harbor mutations in *THRα* gene requires further investigation.

The TRAb-negative patients with hyperthyroidism without exophthalmos or anterior tibial myxedema should be highly suspected for the presence of this disease. Pituitary TSH adenoma should be excluded before making the final diagnosis. Family histories and negative pituitary MRI will benefit for the diagnosis of THRS.^[15]^ Without doubt, genetic testing of THRβ plays an important role in the diagnosis of this disorder. Performing genetic testing of THRβ in suspected patients with THRS and their kindred can facilitate the early diagnosis and correct treatment, which is especially important in the therapy of affected infants and children.

Currently, the treatment of patients with THRS depends on their clinical symptoms.^[1,38]^ If the patient can compensate the organ resistance by increasing endogenous thyroid hormone, intervention is not essential; if hypothyroidism occurs, especially in infants and children, thyroid hormone should be supplemented in time. In cases of patients with selective resistance accompanied by hyperthyroidism, oral administration of triiodothyroacetic acid is preferred as a precise medicine for PRTH, as it suppresses TSH secretion without exacerbating thyrotoxicosis. However, at present, this medication is not available in Mainland China. Patients with symptoms, such as increased heart rate, palpitation, and shortness of breath can take β-receptor blockers. It should be noted that it is the clinical symptoms not the thyroid hormone levels that determine the efficacy of the treatment.

In our study, the diagnosis of PRTH was confirmed by the clinical features, laboratory findings, and gene mutation analysis. And A317T mutation in our patients is the first reported mutation in *THRβ* gene in a Chinese pedigree. The limitations in our study are that owing to the availability of triiodothyroacetic acid, we did not know the treatment effect of this medicine, although we stopped the oral administration of methimazole and administered β-receptor blockers. However, the clinical symptoms of our patients were greatly relieved after the administration of β-receptor blockers. And warming of contradictions to antihyperthyroid agents, isotope therapy, and thyroid surgery was given to the patients.
